# Anti‐Müllerian Hormone as a Marker for Castration Status and Fertility in Bulls and Oxen

**DOI:** 10.1111/rda.70196

**Published:** 2026-03-17

**Authors:** Doreena Gilg, Christiane Otzdorff, Yury Zablotski, Holm Zerbe, Beate Walter

**Affiliations:** ^1^ Veterinary Clinic Gessertshausen Altano GmbH Gessertshausen Germany; ^2^ Small Animal Clinic at the Centre for Clinical Veterinary Medicine, Faculty of Veterinary Medicine Ludwig‐Maximilians‐University Munich Germany; ^3^ Clinic for Ruminants With Ambulatory and Herd Health Services Ludwig‐Maximilians‐University Munich Oberschleissheim Germany

**Keywords:** AMH, bull fertility, castration status, fertility indicator, sperm quality, testosterone

## Abstract

Anti‐Müllerian hormone (AMH) has been established as an indicator of castration status and fertility in several species. However, its applicability in postpubertal bulls and oxen has not yet been thoroughly investigated. This study examined 73 bulls aged between 12 and 107 months and 37 oxen older than 12 months. Blood serum samples were collected from all animals for AMH measurement using a commercial chemiluminescent immunoassay. Additionally, testosterone concentrations were assessed in 32 oxen and 71 bulls using mass spectrometry. In bulls, correlations between AMH levels and sperm concentration, motility and morphology were analysed. The AMH concentration in oxen (median: 0.01 ng/mL) was significantly lower (*p* < 0.001) than in bulls (median: 4.69 ng/mL). While reduced sperm motility had a significant impact on the AMH concentration (*p* = 0.02), this was not the case for sperm morphology (*p* = 0.31). In addition, in bulls with normal semen parameters, AMH concentration showed a negative correlation with age (*p* = 0.01). In conclusion, AMH (≤ 0.01 ng/mL) serves as a reliable marker for distinguishing oxen from bulls, even in older animals. To our knowledge, this is the first study evaluating AMH as a fertility‐associated biomarker in postpubertal bulls.

## 1 Introduction

Anti‐Müllerian hormone (AMH) is a glycoprotein belonging to the transforming growth factor‐beta (TGF‐β) family (Cate et al. 1986). It is secreted by Sertoli cells in the foetal testes and induces the regression of the Müllerian ducts in male individuals. In contrast, in females, the absence of AMH allows the Müllerian ducts to develop into the oviducts, uterus and cranial vagina (Schnorr et al. 2019). In males, AMH is secreted at high levels from testicular differentiation until puberty, after which secretion declines significantly (Josso et al. 2001). AMH has been extensively studied as a fertility marker in cows. Ultrasound examinations have demonstrated that AMH concentrations correlate with the number of antral follicles and can therefore predict the ovarian response to superovulation protocols (Singh et al. 2004). Since AMH is produced by granulosa cells, several studies have investigated the relationship between serum AMH concentrations and superovulation outcomes. Cows with higher AMH concentrations show a better response to superovulation protocols (Rico et al. 2011; Souza et al. 2015). Additionally, higher AMH serum concentrations have been associated with increased pregnancy rates and a reduced risk of pregnancy loss between days 30 and 65 (Ribeiro et al. 2014). Furthermore, a single AMH measurement in heifers has been shown to provide valuable insights into their reproductive potential and overall productive lifespan (Jimenez‐Krassel et al. 2015).

In contrast to the extensive data available for female cattle, information on the role of AMH in bulls is scarce. One study has investigated AMH as an indicator of castration status in male calves. In that study, castration was performed at 8 weeks of age, and AMH levels have been measured between 16 and 48 weeks in castrated calves as well as intact controls. The results have shown significantly lower AMH concentrations in castrated calves compared to intact ones at all time points. Moreover, AMH concentrations in intact calves have declined upon reaching puberty between 32 and 40 weeks of age (Scarlet et al. 2017). Similar studies in dogs, cats and horses have consistently shown significantly higher AMH concentrations in intact males compared to castrated individuals (Axnér and Ström Holst 2015; Claes et al. 2013; Themmen et al. 2016). In dogs, an additional testosterone measurement has been reported to improve the diagnostic sensitivity of AMH testing (Themmen et al. 2016). In male horses, AMH concentrations in colts with normal and abnormal testicular development have been comparable within the first 2 years of life. However, after this period, colts with abnormal testicular development have exhibited higher AMH concentrations than their healthy counterparts (Scarlet et al. 2018). Significantly elevated AMH levels have also been reported in cryptorchid male dogs and in dogs with other disorders of sexual development (Hornakova et al. 2017; Walter et al. 2022).

In humans, reduced sperm concentration, total sperm count and motility have been associated with lower AMH levels (Holt et al. 2023). Conversely, some studies have reported increased AMH concentrations in individuals with abnormal sperm morphology (Aksglaede et al. 2018). Similar findings have been observed in male dogs, where a higher percentage of sperm head abnormalities and a reduced total sperm count have been correlated with higher AMH concentrations (Domain et al. 2022; Hallberg et al. 2024). Furthermore, in male dogs, sperm motility and the proportion of morphologically normal sperm decrease with age, although no correlation between AMH concentration and age has been detected (Hallberg et al. 2024). In contrast, studies in men have demonstrated a decline in AMH concentrations with increasing age (Aksglaede et al. 2010; Chong et al. 2013; Ramezani Tehrani et al. 2017).

However, no studies to date have examined the relationship between serum AMH concentration and sperm motility, concentration or morphology in bulls. This study aimed to evaluate serum AMH and testosterone concentrations as markers of castration status in postpubertal oxen. Furthermore, it investigated the association between AMH levels and semen quality parameters (motility, concentration and morphology) in bulls and examined age‐related changes in AMH concentrations.

## 2 Materials and Methods

73 bulls of different breeds (Fleckvieh, Limousin, Brown Swiss, Belgian Blue) from four artificial insemination (AI) stations and 37 oxen of the breed Fleckvieh from a single fattening unit were included in this study. All of the oxen were between 12 and 22 months old, the exact age was not known. The bulls had a median age of 28 months (range: 12 to 107 months), only the age of one bull was not known. The bulls were divided into two groups. 42 animals were classified as fertile with a sperm motility of 70% or higher and a sperm concentration of 600.000 Mio/mL or higher and 31 animals were classified subfertile with a sperm motility under 70% and sperm concentration under 600.000 Mio/mL. The classification was performed according to the regulations of the Association of German Cattle Breeders for AI stations. The study was approved by the Ethics Committee of the Faculty of Veterinary Medicine, LMU Munich (control number: 255‐12‐02‐2021). Blood samples were collected at the vena caudalis media or vena jugularis externa. Serum samples were allowed to clot for at least 30 min, then centrifuged (Hettich Eba 3S) by 1500 *g* for 10 min before the supernatant was filled in 1.5 mL tubes and frozen at −80°C until measurement.

### 2.1 Anti‐Müller Hormone and Testosterone Measurement

The 110 serum samples were sent chilled to a commercial laboratory (Laboklin GmbH & Co KG, Bad Kissing, Germany) for hormone measurements. AMH was measured once with an electrochemiluminescence immunoassay (Cobas Elecsys AMH Plus) on a Cobas 8000 (Roche) validated for cows (Koca et al. 2023, 2024) according to the manufacturer's instructions. The sensitivity of the test system is 0.01 ng/mL with a minimum detection limit of 0.01 ng/mL and a maximum detection limit of 23 ng/mL. The inter‐assay coefficient of variation is between 1.0% and 1.8% and the intra‐assay coefficient is between 2.7% and 4.4%, as supplied by the manufacturer. In 104 serum samples testosterone measurement was performed with liquid chromatography mass spectrometry, an in‐house developed test system from the commercial laboratory (Laboklin GmbH & Co. KG). Six samples didn't contain enough serum for an additional testosterone measurement. This method is validated for matrix serum and is therefore independent of the species. The minimum detection limit of testosterone is 0.02 ng/mL and the upper detection limit 50.0 ng/mL. The intra‐assay coefficient of variation is 8.20% and the inter‐assay of variation 10.53%. The reference range for testosterone in sexually intact bulls is > 1 ng/mL given by the manufacturer.

### 2.2 Morphological Sperm Evaluation

During routine semen collection in the 73 bulls, a volume of 10 μL of the ejaculate was collected and preserved in 1 mL formol citrate solution and chilled. After collecting all samples, two smears of each sample were prepared for morphological evaluation. Therefore, one drop of the mixture was applied on a slide and smoothed out. Morphological evaluation of 200 sperms of each sample was performed by two examiners with light microscopy at 1000 magnification with oil by manual counting. The alterations were numerically registered and the result was the median value of four examinations in total. Alterations of the acrosome, the head, the mid piece and the tail, as well as lose heads and proximal and distal cytoplasmic droplets were taken into account. Classification of the bulls was performed according to the German regulations (Bundesverband Rind und Schwein 2023) in a fertile group with < 20% morphological abnormal sperm cells and a subfertile group with 20% or more morphological abnormal sperm cells.

### 2.3 Statistical Analyses

Statistical analyses were performed using R version 4.4.0 (2024‐04‐24). A minimum sample size of 26 animals per group was calculated to achieve 80% power with an effect size of 0.8 (two‐sample *t*‐test). To account for potential dropouts, 10% additional animals were included, resulting in 29 animals per group. Normality was assessed using the Shapiro–Wilk test; non‐normally distributed data were analysed with the Mann–Whitney *U* test. These analyses evaluated associations between AMH concentration and castration status, semen evaluation, spermatological alterations, as well as between testosterone concentration and castration status. Spearman correlation was used to assess relationships between numeric variables (AMH and testosterone, AMH and age). A *p*‐value < 0.05 was considered statistically significant. Results are presented as median (minimum–maximum).

## 3 Results

**FIGURE 1:**
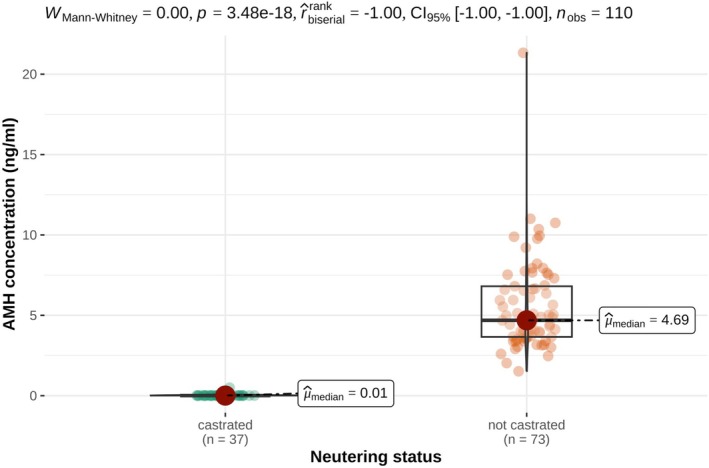
Serum AMH concentrations (ng/mL) in castrated (*n* = 37) and non‐castrated (*n* = 73) male cattle. Individual data points are shown together with boxplots indicating the median (horizontal line), interquartile range (box) and range (vertical line).

**FIGURE 2:**
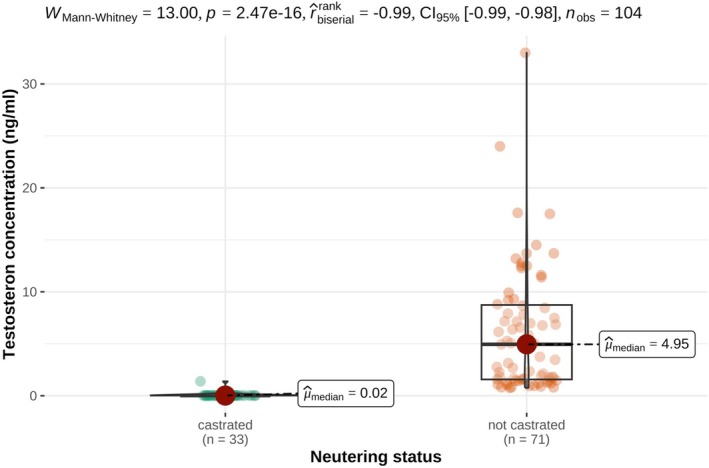
Serum testosterone concentrations (ng/mL) in castrated (*n* = 33) and non‐castrated (*n* = 71) male cattle. Individual data points are shown together with boxplots indicating the median (horizontal line), interquartile range (box) and range (vertical line).

**FIGURE 3:**
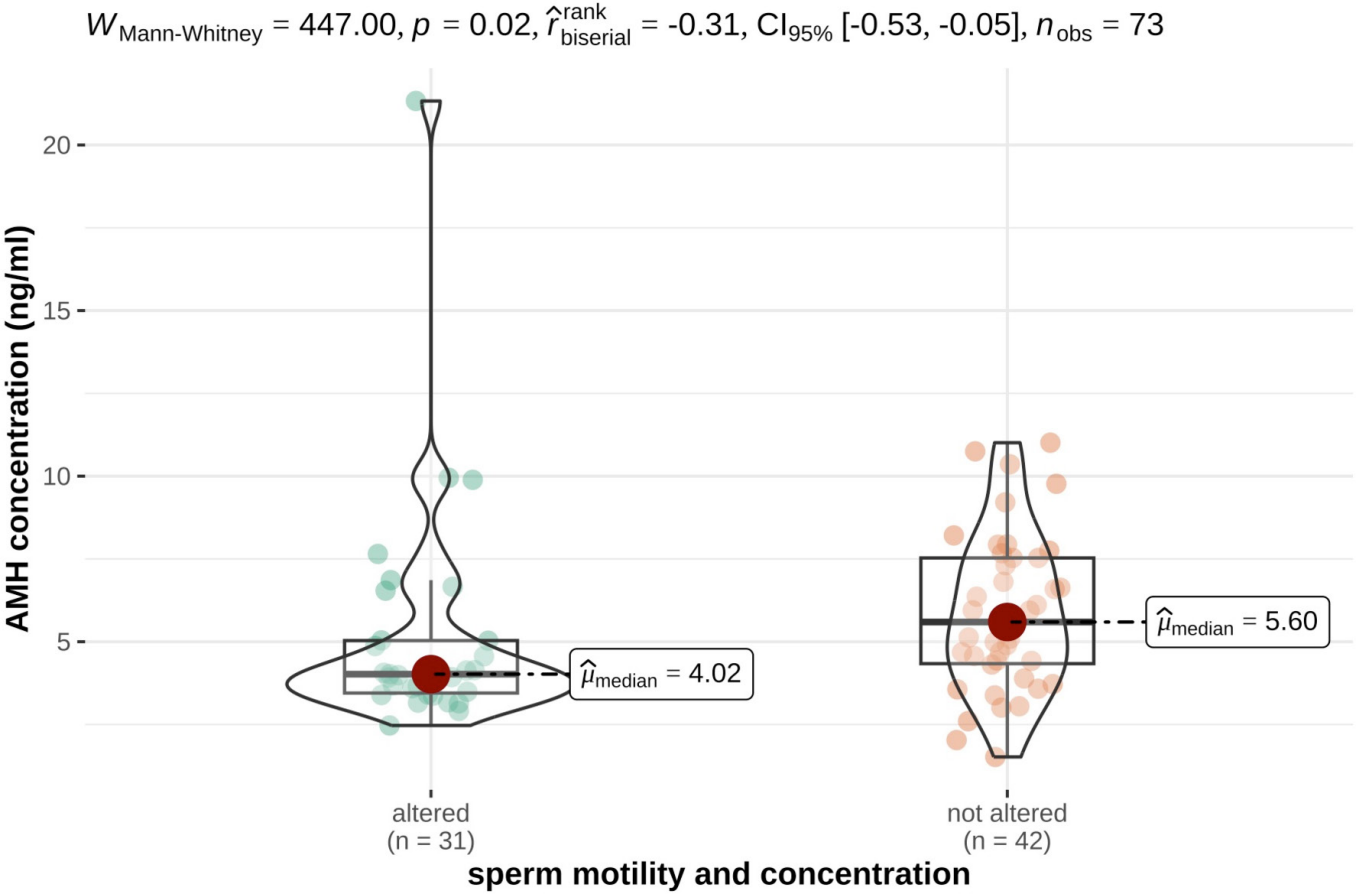
Serum AMH concentrations (ng/mL) in non‐castrated bulls with altered (*n* = 31) and non‐altered (*n* = 42) sperm motility and concentration. Violin plots illustrate the distribution density of the data, overlaid with boxplots indicating the median (horizontal line), interquartile range (box) and range (vertical line). Individual animals are shown as dots.

**FIGURE 4:**
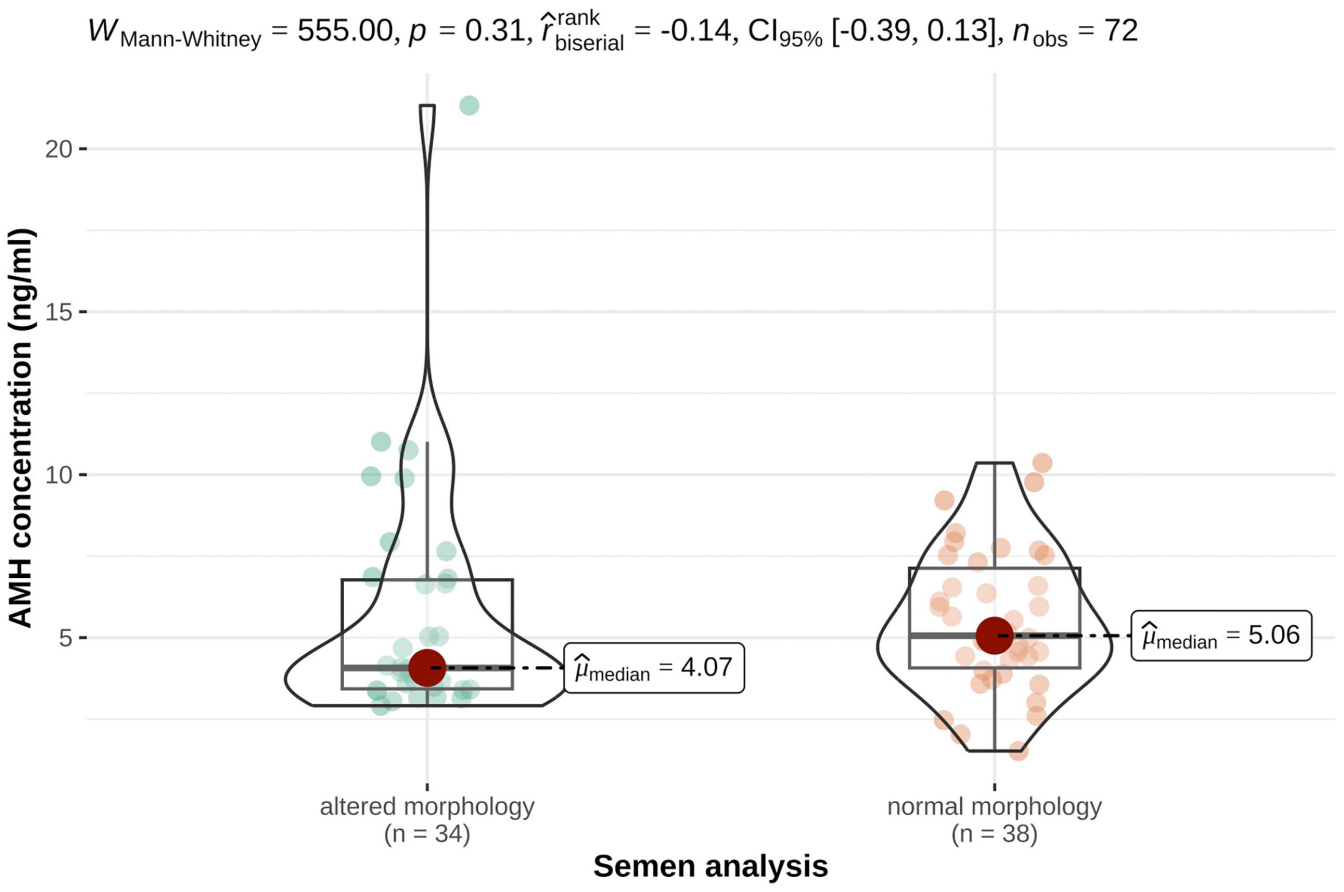
Serum AMH concentrations (ng/mL) in non‐castrated bulls with altered morphology (*n* = 34) and normal morphology (*n* = 38). Violin plots illustrate the distribution density of the data, overlaid with boxplots indicating the median (horizontal line), interquartile range (box) and range (vertical line). Individual animals are shown as dots.

AMH and testosterone concentrations differed significantly between oxen and bulls as presented in Tables 1 and 2 and illustrated in Figures 1 and 2. One ox exhibited markedly higher AMH and testosterone concentrations compared to the remaining oxen. The AMH concentrations of the bulls were between 1.52 and 11.01 ng/mL; only one bull had an AMH concentration of 21.33 ng/mL. In bulls, AMH concentrations were associated with sperm motility and concentration but not with sperm morphology as shown in Table 1 and Figures 3 and 4.

**TABLE 1:** Median, minimum and maximum AMH values in bulls and oxen and in bulls depending on their sperm motility and concentration as well as sperm morphology.

Comparison groups	AMH in ng/mL			*p*
	Median	Minimum	Maximum	
**Castration status**				
Oxen (*n* = 37)	≤ 0.001	< 0.001	0.5	< 0.001
Bulls (*n* = 73)	4.69	1.52	21.33	
**Sperm motility and concentration**				
Bulls (*n* = 31) < 70% motility and < 600.000 Mio/mL	4.02	2.47	21.33	0.02
Bulls (*n* = 42) > 70% motility and < 600.000 Mio/mL	5.60	1.52	11.01	
**Sperm morphology**				
Bulls (*n* = 34) > 20% abnormal sperms	4.07	2.47	21.33	0.31
Bulls (*n* = 38) < 20% abnormal sperms	5.06	1.52	10.75	

*Note:* The *p*‐value was determined using the Mann–Whitney *U* test.

**TABLE 2:** Median, minimum and maximum testosterone value in bulls and oxen.

Comparison group	Testosterone in ng/mL			*p*
	Median	Minimum	Maximum	
**Castration status**				
Oxen (*n* = 37)	0.02	≤ 0.02	1.39	< 0.001
Bulls (*n* = 73)	4.95	0.76	33.00	

*Note:* The *p*‐value was determined using the Mann–Whitney *U* test.

Among the bulls with normal sperm motility and concentration, eight had an amount of abnormal sperm cells above 20% and five bulls with reduced sperm motility and concentration had < 20% morphological abnormal sperm cells.

**FIGURE 5:**
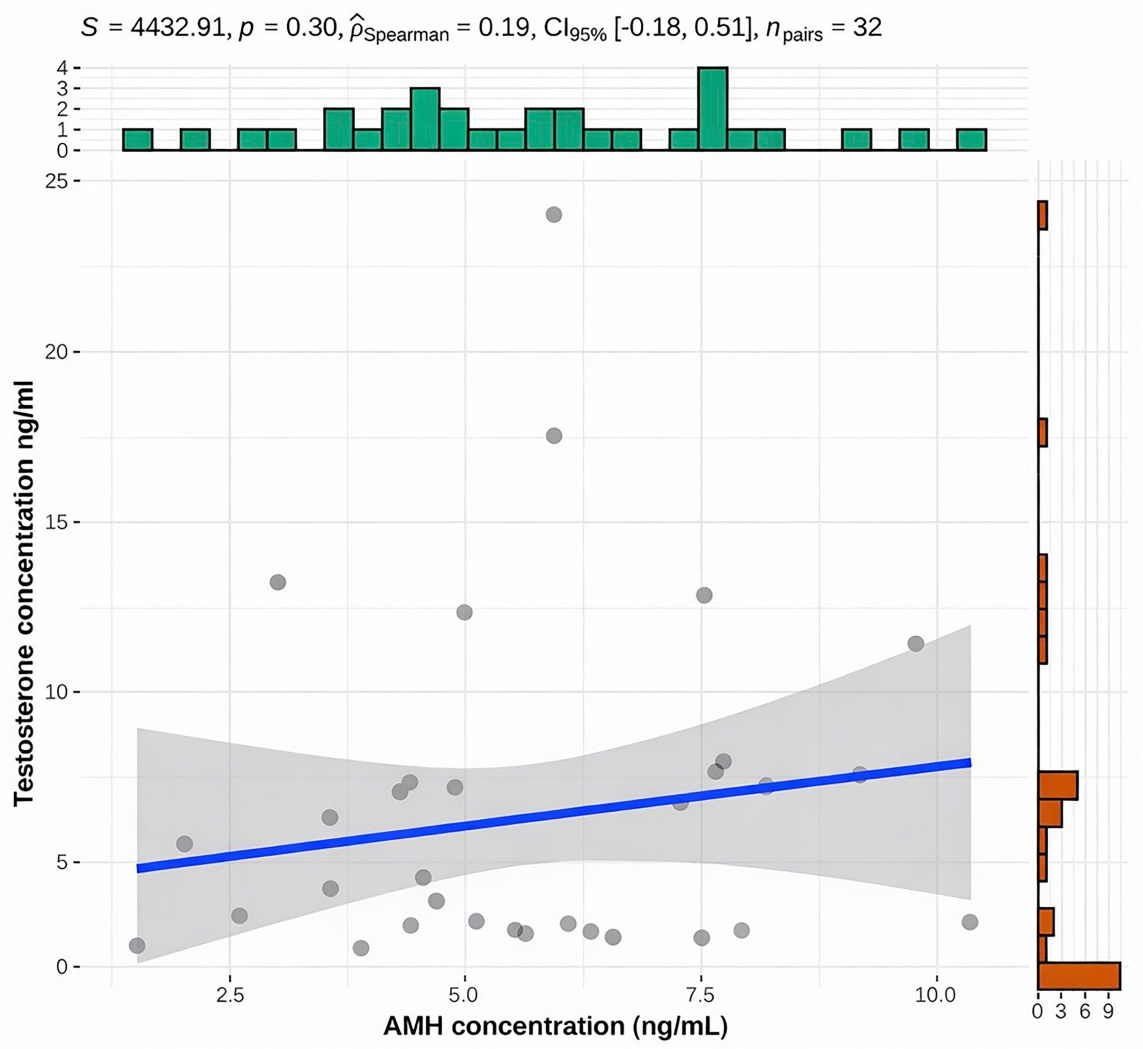
Correlation between AMH (ng/mL) and testosterone concentration (ng/mL) of the bulls with unaltered sperm motility, concentration and morphology (*n* = 32). Individual animals are shown as dots together with a fitted linear regression line and the corresponding 95% confidence interval (shaded area). Marginal histogram depicts the distribution of AMH concentrations (top) and testosterone concentrations (right).

**FIGURE 6:**
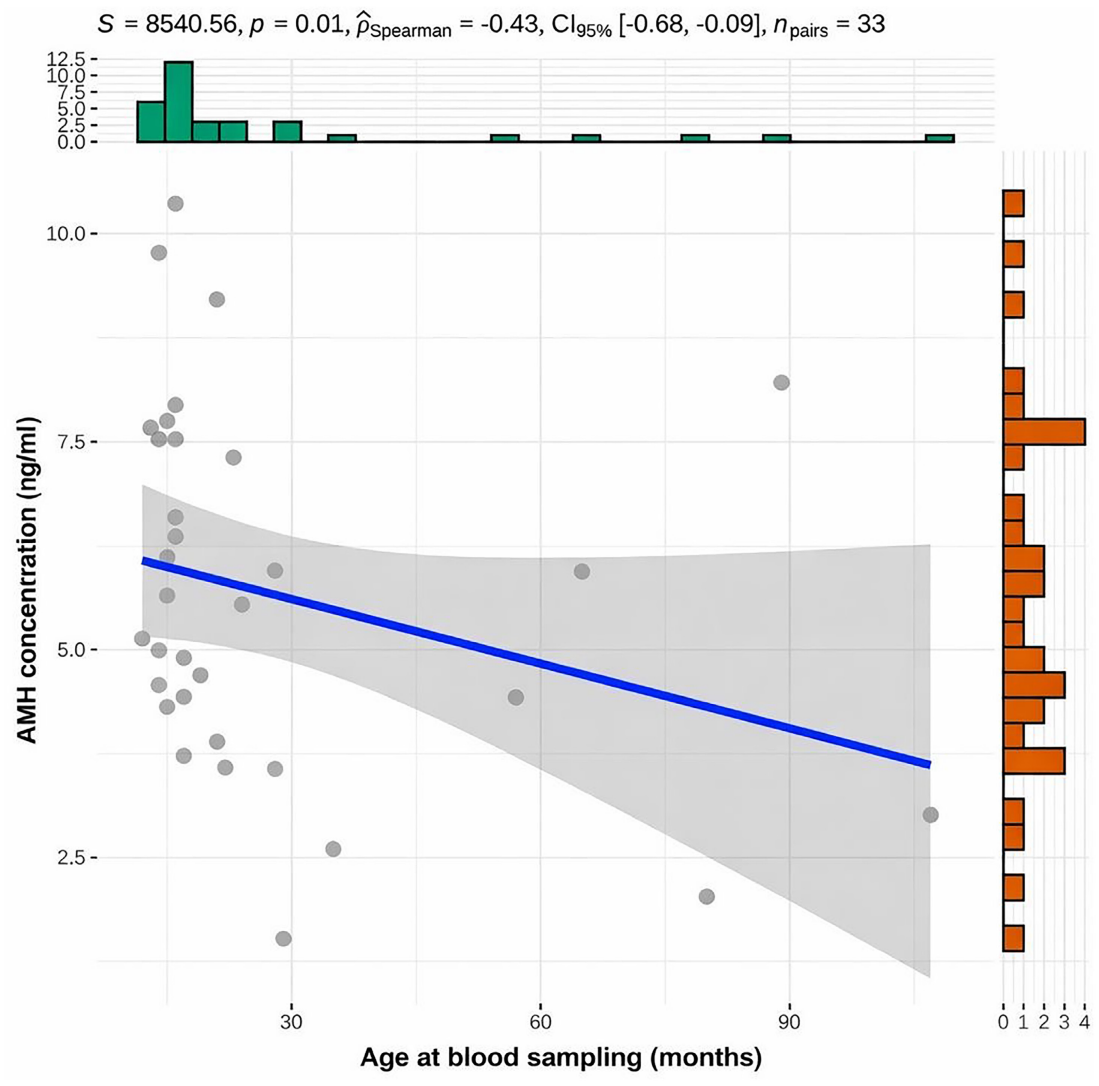
Correlation between AMH concentration (ng/mL) and age of the bulls at blood sampling (months) with unaltered sperm motility, concentration and morphology (*n* = 33). Individual animals are shown as dots, with a fitted linear regression line and the corresponding 95% confidence interval (shaded area). Marginal histograms illustrate the distribution of age at blood sampling (top) and AMH concentrations (right).

As shown in Figure 5 no correlation between AMH and testosterone concentrations was found in bulls with normal semen quality. However, age was negatively correlated with AMH concentration in bulls with unaltered sperm motility, concentration and morphology, as depicted in Figure 6.

## 4 Discussion

This study showed for the first time that AMH serum concentration can be used to prove castration success in male cattle older than 12 months and not only up to 48 weeks as previously described (Scarlet et al. 2017). In this context, no overlap in hormone concentrations was observed between intact and castrated animals. These findings indicate that a single AMH measurement is highly reliable to assess castration status and to distinguish intact and castrated individuals. The use of AMH as a castration marker has also been reported in tomcats, dogs and stallions (Axnér and Ström Holst 2015; Claes et al. 2013; Themmen et al. 2016) as well as in queens, bitches and female rabbits (Böhmer et al. 2022; Place et al. 2011).

Furthermore, this study demonstrated that testosterone concentrations differed significantly between oxen and bulls. However, overlap in testosterone concentrations between the groups was observed. Seven intact animals exhibited concentrations below the reference range defined for intact animals, while two animals from the oxen group showed measurable testosterone concentrations. One of these fell within the reference range for intact animals. A possible explanation for these fluctuations of the testosterone levels in the animals of this study is the pulsatile secretion of this hormone which has been described (Brito 2021). However, the ox with the elevated testosterone level also exhibited a measurable AMH concentration, which may suggest that the bloodless castration was not entirely successful and that some degree of testicular activity persisted. This interpretation is supported by the observation that several oxen phenotypically resembled bulls during blood sampling. Unfortunately, confirmation of this assumption through subsequent clinical examination was not possible, as individual animals could not be identified due to the absence of ear tag documentation.

A significant positive correlation between AMH concentration and sperm motility and sperm concentration was observed in the bulls of this study. Unlike that, no correlation between AMH concentration and sperm morphology was detected. Therefore, AMH may provide complementary information in the evaluation of fertility in bulls. In contrast, studies in dogs have demonstrated a negative correlation between serum AMH levels and sperm motility and morphology (Domain et al. 2022; Hallberg et al. 2024), whereas in human medicine, findings differ on this matter. One study has found that significantly elevated serum AMH concentrations are associated with a lower percentage of morphologically normal sperm (Aksglaede et al. 2018). Other studies have observed that in infertile men with impaired gonadal function, AMH levels, sperm motility, sperm concentration and total sperm count were reduced (Holt et al. 2023). Also, with respect to azoospermia, findings regarding AMH are inconsistent. While men with azoospermia exhibit significantly lower AMH concentrations compared with individuals with normal semen parameters, dogs with azoospermia or oligozoospermia display significantly elevated AMH concentrations (Walter et al. 2026; Zec et al. 2011). Given the variability of the reported findings, studies with larger cohorts are necessary to assess the utility of AMH as a fertility marker for selected semen parameters.

In the present study no correlation between testosterone and AMH concentration was found in bulls with normal semen parameters. A very likely explanation for the lack of correlation is the age of the animals examined, as a negative correlation has been described predominantly during puberty in boys, cattle, male dogs and mice (Alexander et al. 2024; Rey and Josso 1996; Rey et al. 1999; Rota et al. 2002). In horses this inversed correlation between AMH and testosterone has been shown with examinations before and after puberty (Claes et al. 2013). Similarly to the present study, no correlation between AMH and testosterone has been reported in postpubertal male alpacas (Ciccarelli et al. 2018). Another possible explanation for the lack of correlation may be the single‐time blood sampling since testosterone is secreted in a pulsatile manner (Brito 2021; Senger 2012). Further studies incorporating repeated blood sampling or, alternatively, non‐invasive assessment of testosterone metabolites in faeces could help to clarify this issue (Auer et al. 2020; Martin 1966; Mooring et al. 2004).

A negative correlation between AMH levels and the age of postpubertal fertile bulls, with AMH concentrations decreasing with age, has been found in this study. In human medicine, studies show that AMH levels remain relatively constant in adulthood (Aksglaede et al. 2010). However, there is one study indicating a decline in serum AMH concentration with increasing age also in humans (Ramezani Tehrani et al. 2017). A decline in AMH concentration with advancing age has also been described in bitches (Hollinshead et al. 2017). Nevertheless, in this context, AMH concentrations exhibit substantial variability and bulls of the same age exhibit differences in AMH concentration. A possible explanation for the observed variation in AMH concentrations among age‐matched young adults is that despite identical chronological ages testicular maturation may vary. Given the limited age range and the small number of older animals included in this study, the association between AMH concentration and age warrants further investigation.

One bull in this study exhibited an exceptionally high AMH concentration. Elevated AMH concentrations have been reported in prepubertal individuals, including boys, tomcats, dogs, stallions and bull calves, where immature testes are characterised by high AMH secretion (Alexander et al. 2024; Claes et al. 2013; Josso et al. 1990; Rota et al. 2002). In addition, significantly increased AMH serum levels have been described in dogs with experimentally induced testicular atrophy, as well as in cryptorchid dogs and stallions (Balogh et al. 2021; Claes et al. 2013; Prapaiwan et al. 2023). Furthermore, Sertoli cell tumours in dogs and men have been associated with elevated AMH concentrations (Holst and Dreimanis 2015; Rey et al. 2000). The bull examined in the present study was at least 12 months old and housed at an artificial insemination station, making cryptorchidism and testicular neoplasia unlikely. Nevertheless, this bull exhibited reduced sperm motility and concentration as well as a markedly increased proportion of morphologically abnormal sperm, which could indicate delayed testicular maturation or degenerative changes. A significant increase in AMH concentration due to testicular degeneration has been described in male dogs (Walter et al. 2026), but this relationship has not yet been systematically investigated in bulls. In addition, potential antibody interference should be considered when individual results appear as outliers, as previously described in dogs and cats (Bergman et al. 2019, 2018). Notably, such interference has not been demonstrated in horses (Dong et al. 2021); moreover it seems unlikely that antibody interference alone would account for an increase in AMH concentration exceeding twofold, given that only minor increases were observed in dogs and cats (Bergman et al. 2019, 2018). However, immunoassay interference is considered sporadic and unpredictable, and its presence cannot be excluded solely based on the magnitude of deviation. Therefore, although antibody interference appears unlikely, it cannot be definitively ruled out in individual cases. Further studies with more affected bulls and pathohistological examination of the testicles would be required to examine the reason of significantly increased AMH concentration in postpubertal bulls further.

In summary, this study demonstrates a significant association between serum AMH and testosterone concentrations and the castration status of male cattle older than 12 months. Castrated animals showed markedly lower hormone concentrations, with median AMH values below 0.01 ng/mL and median testosterone values below 0.05 ng/mL, whereas intact animals showed substantially higher values. These findings suggest that low serum concentrations of AMH and testosterone may be useful indicators of successful bloodless castration under the conditions of this study. The observed hormone ranges should be interpreted as population‐based reference values rather than strict diagnostic cut‐off thresholds. Intermediate concentrations showed overlap between castrated and incompletely castrated animals, indicating that combined hormone assessment and clinical examination are advisable in equivocal cases. In addition, AMH concentrations were positively correlated with sperm motility and concentration, but not with sperm morphology, supporting its potential role as an indicator of testicular function.

Key limitations included small subgroup sizes for fertility analyses, single‐time‐point hormone measurements and the lack of longitudinal or pathological validation. Therefore, while AMH appears to be a promising adjunct parameter in bovine reproductive assessment, further studies with larger cohorts and repeated sampling are required to define its diagnostic and fertility‐related applicability.

## Author Contributions

Conceptualization: Beate Walter, Christiane Otzdorff, Holm Zerbe. Methodology and statistical analysis: Yury Zablotski. Investigation: Doreena Gilg. Writing – original draft preparation: Doreena Gilg, Beate Walter. Writing – review and editing: Christiane Otzdorff, Holm Zerbe. Supervision: Christiane Otzdorff, Holm Zerbe, Beate Walter.

## Funding

The authors have nothing to report.

## Conflicts of Interest

The authors declare no conflicts of interest.

## Data Availability

The data that support the findings of this study are available from the corresponding author upon reasonable request.
